# P22 Rare case of *Salmonella houtenae*-associated osteomyelitis and discitis in an older patient with non-specific symptoms: a management challenge

**DOI:** 10.1093/jacamr/dlaf230.029

**Published:** 2025-12-04

**Authors:** Sanan Arshad

**Affiliations:** University Hospital Birmingham, Birmingham, UK

## Abstract

**Background:**

Salmonella is a significant zoonosis in developing countries, causing gastroenteritis, septicaemia and typhoid fever, typically via contaminated food or water. *Salmonella enterica* subsp. *houtenae* is common in reptiles and can cause severe human infections.^1’^This case highlights the diagnostic and management challenges posed by this rare and potentially life-threatening subspecies.

**Case presentation:**

A 78-year-old woman with type 2 diabetes, hypertension, heart failure and atrial fibrillation presented with leg oedema, cough and hypoxia. She was treated for pneumonia and decompensated heart failure but re-presented following a fall and faecal incontinence. Examination showed reduced lower limb power. She denied recent takeaway food, reptile/rodent contact, prosthetic material, or gastrointestinal symptoms suggestive of Salmonella infection. Inflammatory markers were raised, and blood cultures grew a non-typical *Salmonella* species. MRI revealed T12–L1 osteomyelitis/discitis. Genome sequencing confirmed *Salmonella houtenae*, a rare subspecies seldom reported in humans. The isolate was susceptible to azithromycin, cotrimoxazole, ciprofloxacin, ceftriaxone and meropenem. Three consecutive blood cultures confirmed the organism, while stool cultures remained negative. She was initially treated with co-trimoxazole, then IV ceftriaxone, due to which she developed confusion. With persistent inflammation and radiological progression, azithromycin was added, followed by high-dose ciprofloxacin with ceftriaxone. Due to significant comorbidities, she was deemed unfit for neurosurgical or spinal intervention. Her condition stabilized with antimicrobial therapy, and she was discharged to an enhanced care unit once CRP improved.

**Discussion:**

*Salmonella houtenae* is a rare subspecies, accounting for less than 1% of *Salmonella* strains.^2^ Only one human case has been reported previously, involving empyema in a 70-year-old patient with chronic TB.^2^ We present an elderly female with *S. houtenae* infection causing osteomyelitis/discitis. *Salmonella* bacteraemia in older patients without known exposure is uncommon and likely results from contact infection or bacterial translocation from intestinal colonization.^3^ The gastrointestinal tract remains the primary entry point, encompassing typhoidal salmonellosis (*Salmonella* Typhi*, Salmonella* Paratyphi A) and non-typhoidal salmonellosis (NTS) from other *Salmonella* species.^4,5^ While typhoidal and paratyphoidal *Salmonella* are well documented as causes of bacteraemia, this case underscores the significance of *S. houtenae* intestinal colonization leading to bacteraemia and bone involvement in elderly patients, especially with comorbidities. Repeated blood cultures consistently growing *S. houtenae*, in the absence of other pathogens, highlight the need to recognize this rare subspecies as a potential human pathogen.

**Conclusions:**

*Salmonella* as an aetiological agent in osteomyelitis is essentially rare. It is the causative organism in 0.45% of osteomyelitis, and *Salmonella* osteomyelitis itself accounts for as few as 0.8% of all Salmonella infections.^6^ We describe the first reported case of *S. houtenae* osteomyelitis. The patient’s advanced age and comorbidities increased susceptibility to non-typhoidal *Salmonella*. This case emphasizes recognizing rare *Salmonella* subtypes in humans and the value of molecular diagnostics for accurate identification and optimal antibiotic therapy.
